# High quality, patient centred and coordinated care for Alstrom syndrome: a model of care for an ultra-rare disease

**DOI:** 10.1186/s13023-015-0366-y

**Published:** 2015-11-24

**Authors:** Stephanie Van Groenendael, Luca Giacovazzi, Fabian Davison, Oliver Holtkemper, Zexin Huang, Qiaoying Wang, Kay Parkinson, Timothy Barrett, Tarekegn Geberhiwot

**Affiliations:** London School of Economics, London, UK; Oliver Holtkemper, London, UK; Alstrom UK, London, UK; Institute of Cancer and Genomic Sciences, University of Birmingham, London, UK; Department of Endocrinology, University Hospital of Birmingham, London, UK

**Keywords:** Alstrom syndrome, Rare disease, Multidisciplinary clinics, National service

## Abstract

**Background:**

Patients with rare and ultra-rare diseases make heavy demands on the resources of both health and social services, but these resources are often used inefficiently due to delays in diagnosis, poor and fragmented care. We analysed the national service for an ultra-rare disease, Alstrom syndrome, and compared the outcome and cost of the service to the standard care.

**Methods:**

Between the 9th and 26th of March 2014 we undertook a cross-sectional study of the UK Alstrom syndrome patients and their carers. We developed a semi-structured questionnaire to assess our rare patient need, quality of care and costs incurred to patients and their careers. In the UK all Alstrom syndrome patients are seen in two centres, based in Birmingham, and we systematically evaluated the national service and compared the quality and cost of care with patients’ previous standard of care.

**Results:**

One quarter of genetically confirmed Alstrom syndrome UK patients were enrolled in this study. Patients that have access to a highly specialised clinical service reported that their care is well organised, personalised, holistic, and that they have a say in their care. All patients reported high level of satisfaction in their care. Patient treatment compliance and clinic attendance was better in multidisciplinary clinic than the usual standard of NHS care. Following a variable costing approach based on personnel and consumables’ cost, our valuation of the clinics was just under £700/patient/annum compared to the standard care of £960/patient/annum. Real savings, however, came in terms of patients’ quality of life. Furthermore there was found to have been a significant reduction in frequency of clinic visits and ordering of investigations since the establishment of the national service.

**Conclusions:**

Our study has shown that organised, multidisciplinary “one stop” clinics are patient centred and individually tailored to the patient need with a better outcome and comparable cost compared with the current standard of care for rare disease. Our proposed care model can be adapted to several other rare and ultra-rare diseases.

## Background

Alstrom syndrome (AS) is an ultra- rare inherited genetic disorder affecting less than one in a million people globally. AS is characterised by progressive visual impairment usually present within the first year of life; sensorineural hearing loss often in the first decade of life; and cardiomyopathy with progression to heart failure either in infancy or in adolescence. Obesity with all its metabolic consequences such as diabetes, dyslipidaemia and hypertension develops in early childhood. Additionally, a large proportion of AS patients suffer from kidney and liver disease. There is as yet no disease specific treatment available and the disease usually progresses to death in childhood (from cardiac failure) or early adulthood (cardiac or renal failure) where survival beyond the age of 50 is rare [1]. These combinations of pathologies mean that patients require appointments with multiple subspecialists, take many medications, and are frequently hospitalised.

The current model of health care in Europe and beyond is well designed to cater for patients with common conditions. However, these care delivery models are not suited for patients with complex multisystem diseases with health needs that cross subspecialties. As with many rare diseases, awareness of AS amongst the medical community is very low and most physicians caring for these patients may only see one or two patients throughout their career. The medical profession is therefore unfamiliar with the condition, and has minimal specific training, resulting in a feeling of unpreparedness to provide optimal care. This unfamiliarity leads to numerous misdiagnoses, mistreatment and even harm to patients.

Most patients with rare diseases have complex multisystem medical needs requiring patient-centred multidisciplinary clinics. The lack of patient/disease tailored health services has led to the development of patient self-support groups in the rare disease community to help navigate families through the existing health care system [2]. In 2000, Alstrom Syndrome UK (ASUK) was established by a handful of families, who then persuaded their physicians to set up a multidisciplinary clinic (MDC) in a hotel funded by the charity [3]. Five years later, ASUK’s contribution to providing care to their members was recognised by the UK department of health and a national Alstrom service was established for both children and adult patients. Ten years later, the National Health Service (NHS) England highly specialised Alstrom’ service is the only organised MDT service for AS sufferers in the world and accepts patient referrals from as far away as Australia.

The provision of personalised multidisciplinary care by an expert centre has to be cost effective. In consequence, in collaboration with the London School of Economics we undertook a cost benefit analysis of the NHS England Alstrom syndrome national service. This study aimed to identify the costs and benefits of running a patient centred highly specialised clinic for an ultra-rare disease and share best practices.

### Study background

#### Setting

In the UK, there are two nationally commissioned highly specialised clinics for AS based in Birmingham; one is specialised for children and the other for adults. Both hospitals provide multidisciplinary team (MDT) annual review clinics, each for respective age groups.

The patient support group ASUK is recognised by the department of health as an equal partner and it receives direct funding from NHS-England to provide support to the patients and their families during their clinic visit. These clinics have personalised shared care arrangements with the local care providers.

The clinics occur every three months and last 2 and half days as follows: Day one takes place in a local hotel and days 2 and 3 are conducted in a hospital setting.

#### Day 1: Round table discussion and psychological support

The adult patients arrive at their hotel by mid-afternoon, receive a detailed personalised schedule during their stay in Birmingham and further explanation is given as required, and after a brief rest they are invited to attend a round table meeting with the family support group and the clinical team. The first part of the meeting is a group discussion: meeting other patients and families, sharing patients’ experiences, understanding what treatment methods were particularly effective and which were not, and how other families overcome challenges potentially faced by an individual patient. Day 1 also includes asking the patient’s view about the NHS-specialist clinics; what is working for them, and what changes they would like to see in the future. This round table patient led discussion also consists of a discussion of progress in AS research and new developments, and any future potential benefit. The second part of the session consists of psychological support to patients in a private room as required. Accommodation and hotel meeting rooms are all organised and paid for by ASUK. The child and young person patients arrive at their hotel by mid afternoon the day before the clinic, have 24 h blood pressure monitors fitted if required, and participate in an educational event such as making healthy foods, in the parent kitchen area. At the same time, the transition coordinator, dietician, paediatric specialist nurse and doctor are on hand to meet families, answer any questions and allay any anxieties. The following day there is psychological support for families during the clinic, and a round table discussion by young people on what they would like from the clinic.

#### Day 2: Investigations and clinics

Investigations typically consist of blood and urine tests; cardiac magnetic resonance imaging; high resolution chest computerised tomography; abdominal ultrasound; lung function test; ECG; 24 h blood pressure monitoring; urodynamic study; and other tests as deemed appropriate. Many of these tests are individually tailored.

The consultations in day 2 include: Hearing test and adjustment to their hearing aids; exercise and physiotherapy; pharmacy review of patients’ medical treatment and compliance; AS specialist nurse review and additional psychological support.

#### Day 3: MDT clinics

Patients rotate between the various specialists in the clinic as follows:

- Metabolic- consists of metabolic physicians and diabetic specialist nurses. The overall MDT clinics are coordinated by the metabolic team.
- Cardiology- consultant cardiologist and cardiac technician. Echocardiogram in the clinic and review of their clinical progress and optimisation of cardiac treatment.
- Respiratory
- Dietetics
- Ophthalmology
- Bespoke clinics- urology, hepatology, dermatology, ENT, gynaecology, genetics and others as required

At the end of the clinic there is an MDT meeting with all specialist care givers to discuss individual patients and, if required, make further changes to their management. Following this MDT meeting if any of the patients’ care plans change, those patients would be called in one more time and changes would be discussed and agreed with the individual patient.

## Methods

Two separate research pieces were conducted to analyse the effectiveness of the MDC, both from a qualitative and a quantitative standpoint. This involved a survey of all AS patients registered with ASUK in the UK, a cost analysis of the clinical service providers, and a quality of life analysis as follow:

### Survey

Between the 9th and 26th of March 2014 we undertook a cross-sectional study and enrolled 100 % of all AS patients and carers in the UK with a 23 % response rate. Patients and carers were invited for pre-interview discussions and this formed the basis of the structured interviews. The semi-structured interviews were designed to address the socio-economic implications of living with, caring for, and treating patients suffering from AS [5]. The interview design was a semi-structured set up, which was built around open and semi-open questions, providing freedom to the respondent to focus on different subjects and comment on the most relevant issues of their health care [4]. The interviews were conducted either on the telephone or face to face lasting on average 35–60 min.

We undertook measures to improve the reliability (providing consistent measures in comparable situations) and validity (ensuring that answers correspond to the questions that they were intended to measure) of the questionnaires as follow:

- Careful attention was paid to how the survey was constructed: for example, the phrasing of questions was rigorously analysed and refined; spacing of questions and section headings was designed to stimulate understanding and structure the survey to be more effective and intuitive.
- Data validation parameters were implemented into the digital survey (in other words, if a question required a numerical answer, only numeric inputs were accepted) [6].
- All respondents were asked identical sets of questions.
- Interviewers were trained to avoid potential biases affecting responses. Interviewers were provided a script to which they were expected to strictly adhere to.
- All questions needed to be answered in order to progress through the survey.
- Surveys were kept completely anonymous. This was to ensure that all participants were willing to answer questions as accurately as possible without fear or favour.

In our data collection we took the following steps to minimise bias as described by Fowler et al. [7]. To avoid sample frame bias we set up our surveys so that the patient or their caregiver could complete it. For those who were unable to complete the survey due to its format, we offered the option of talking volunteers through the survey and completing them on their behalf. Assistants were briefed to ensure no bias was introduced during this process. Given the low prevalence of the syndrome, we attempted to reach out to all AS sufferers across the UK to avoid human discretion in patient selection. Patient details were obtained from AS UK’s database based on patients’ availability and willingness to participate in this research project. In addition, the survey was set up using an online digital form, which eliminated access problems relating to survey dissemination, completion and collection. To ensure everyone was able to complete the survey we provided support such as telephone assistance to work through the survey with patients who were unable to complete it due to varying sensory losses. We also ensured the survey was compatible with screen reader software such as JAWS, NVDA, ChromeVox, and VoiceOver. We also sought advice from experts in the field of dual sensory losses.

### Cost analysis

To better understand the efficacy of the clinics we undertook to determine whether the cost of their set up and running is feasible for national health care providers. This achieved, direct comparisons between MDT clinical care and standard practices could be drawn. The costing approach can be described as follows. First, a detailed overview of all aspects of the clinics was set up with the help of patient interviews, as described above. In a second step, we conducted a site visit and physician interviews to gain a more detailed (and medical) picture of the exact treatments involved, their layout in the hospital and the respective personnel involved in each practice. Third, we spoke to a finance manager from both care providers who were able to provide valuable insights into the costs involved in care (the cost categories employed by the NHS, and appropriate hourly rates for each activity in the clinic).

Based on our initial interview findings, we therefore formed three cost categories:

1. **Variable direct personnel costs** defined as the hourly rates of specialist physicians (such as a cardiologist), technicians (such as a cardiac technician), nurses (for example for monitoring purposes) and support personnel (for example providing organisational assistance and helping moving patients between clinics).
2. **Consumables** defined as all materials that might be used in a treatment that are non-capital expenses, and would mostly be used only once (such as bandages). In the model, these were taken as a percentage of the personnel costs and adapted to the best of our knowledge depending on the treatment type.
3. **Capital expenses and overhead** divided into two buckets: first, there is the capital equipment to consider that is used to perform the examinations. If this equipment has a useful life of 5-10years, for example, this cost could arguably be allocated to each treatment. However, as there is no direct association between the use of the equipment and their depreciation, this cost was not assigned to the MDC. We believe this is particularly fair as to the best of our knowledge, no additional equipment had to be purchased to run the MDC and all equipment was already available and in use. The same logic applies to overhead costs, such as rent. As no additional facilities had to be rented to run the clinics, this cost is arguably fixed and does not need to be considered in the model, which in theory should only investigate the marginal cost of treatment.

The cost of the standard care was derived from the interview based on the number and speciality of clinics they had attended. We then used the NHS-national tariff to calculate the annual clinics cost.

### Quality of life analysis

We used an additional measure to assess patient quality of life resulting from the use of MDC services. We measured this variable using the Health Utilities Index scales (HUI) that uses the factors of vision, hearing, speech, walking, dexterity, happiness, cognition, and pain in the measure related to good health. This yielded a realistic assessment of AS patient’s quality of life compared to both healthy individuals and other patients suffering from chronic life-limiting diseases.

### Statistical analysis

Interview transcripts and field notes were thematically analysed using a combination of inductive and deductive coding. Descriptive analysis was performed and a two-tailed p-value <0.05 was assumed as statistically significant. Statistical analysis of the two-tailed p-value test was performed using SPSS 17.0 software.

## Results

### Quantitative clinical valuation results

Based on the physician interviews we were able to identify 12 areas of treatment, which we bundled into 12 cost centres. Each cost centre was assigned both its direct variable personnel costs and the cost of consumables (if any). As stated above, capital expenses and overhead were not allocated.

**Fig. 1:**
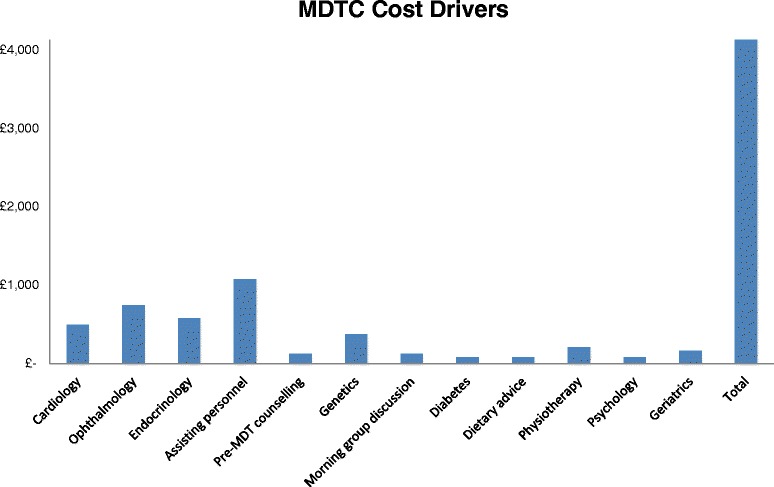
Direct personnel cost of the national multidisciplinary clinics. All costs are in British pounds per clinic for six patients at a time

AS shown in Fig. 1 the total cost for each MDT clinic thereby amounts to GBP 4,138 for a total of 6 patients, or approximately GBP 690 per annum per patient (assuming single annual visits and 6 patient clinics). By far the largest cost category in our model surprisingly turned out be assistant personnel costs – i.e. for nurses involved in the organisation and set up of the MDT clinics. Other major cost drivers (defined as costs contributing more than 10 % of total costs) included experts’ time in the fields of ophthalmology (18 %), endocrinology (14 %) and cardiology (12 %). Nurse and dietician counselling in dietary advice, diabetes and psychological treatment time turned out to be the least expensive elements of the MDT with only 2 % contribution to total cost each.

**Table:** 

Major cost drivers (>10 % of total cost)	Minor cost drivers (<10 % of total cost)
▪ Cardiology (12 %) ▪ Ophthalmology (18 %) ▪ Endocrinology (14 %) ▪ Assisting personnel (26 %)	▪ Pre-MDT counselling (3 %) ▪ Genetics (9 %) ▪ Morning group discussion (3 %) ▪ Diabetes (2 %) ▪ Dietary advice (2 %) ▪ Physiotherapy (5 %) ▪ Psychology (2 %) ▪ Geriatrics (4 %)

The cost analysis of the MDT clinic hereby gives an indicative estimate of the costs involved in the set-up, running and treatments involved in the MDT clinic. As pointed out in the data limitations section, though, much deeper analysis of the respective services involved would have to be conducted to make this figure more realistic.

Our cost assessment of the standard care based on individual specialists clinic cost was estimated to £960/patient/year as per 2014 National Health Service tariff [8].

### Qualitative patient related quality of life results

**Fig. 2:**
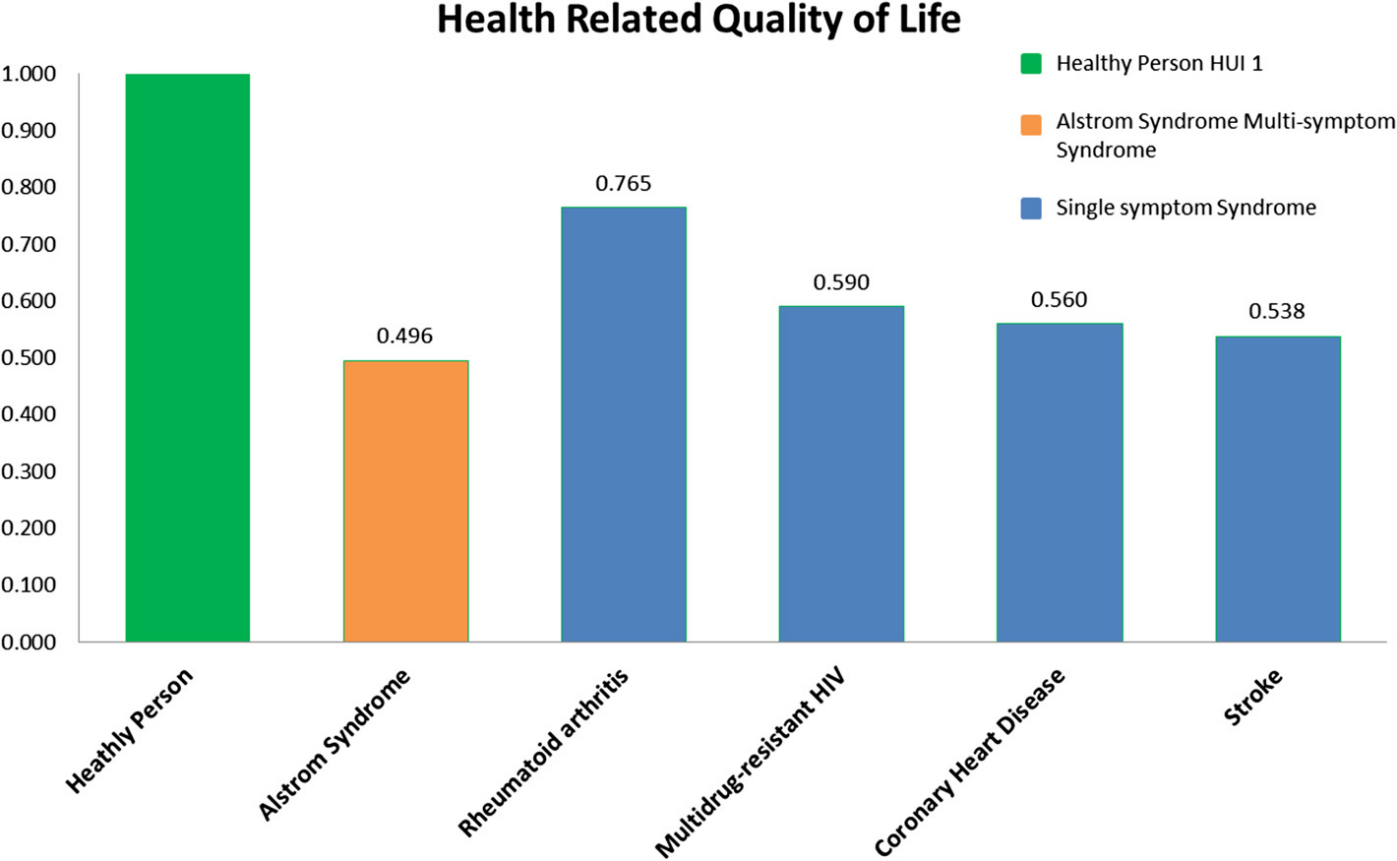
Health related quality of life for Alstrom syndrome patients compared to other chronic diseases

The quality of life analysis, as shown in Fig. 2, demonstrates clearly that patients with AS have a worst quality of life (0.5) of that of a healthy individual and have a lower HUI scale than other common chronic diseases including Rheumatoid arthritis or coronary heart disease.

### Patient satisfaction

Patient has shown a high level of satisfaction in their care delivery, continuity and integration (data not shown).

## Discussion

From our experience, 21^st^ century medical care is tailored to common diseases with relatively high prevalence and has improved the general standard of health care considerably. However, a process designed for common diseases will require further adjustment to cater for patients with rare and ultra-rare diseases. The NHS in the UK has recognised the need for commissioning highly specialised services separately with an establishment of clinical expert centres ranging from 1 to 7 per disease or groups of disease sufferers. Many of these highly specialised services are dedicated to providing care for rare and ultra-rare diseases. The AS service in our centre is the largest centre for this disease in the world

This research has shown that organised, patient centred, multidisciplinary “one stop” clinics are more patient-friendly with better outcomes than the current care model where patients with rare diseases see several specialists at different sites and at different dates. There is no published data to support or refute this. Our study compared the current one stop clinic with their previous standard of care and the results showed a high level of patient satisfaction in their MDT clinics and with several added benefits to their care. We found that our model of patient-centre, knowledge-based rare disease MDT clinics have improved care for patients and knowledge to care givers as follow:

### Patients

- High level of satisfaction with their care: Satisfaction with AS care encompasses care delivery, expectations, attitudes, and disease management. Patients were highly satisfied with communication, approach to addressing their complex need and the time they were given to spend with each specialists. Patients felt their MDT clinic care givers are expert clinicians who are familiar with their conditions. In recent years it has become evident that the patient experience of care is a valuable quality measure to improve care processes [9]. Our study is in agreement with previous evidence that showed team-based care is better than usual care in improving patient satisfaction [10].
- Continuity of care: AS specialist centres provide support to local care givers. If and when challenging health needs arise patients can be referred back to their expert centre as per the shared care pathway. It has been shown that patient care improves considerably when there is a shared care arrangement between local and expert centres [11].
- Certainty: Patients no longer need to suffer from conflicting information from health care providers who are unfamiliar with patients’ disease condition. Patient confidence in the health care system is a prerequisite for patient engagement in their chronic and progressive disease care. The open led discussions in Birmingham concluded that there was a high level of patient confidence towards the MDT clinics. Evidence suggests patient dissatisfaction leads to decreased compliance and poor disease control [12].
- Time efficient: Only two days are required for the MDC visit and 5 more days to see their local care providers per year. The alternative is fragmented care, entailing numerous days wasted seeing different physicians and having numerous and repetitive investigations.
- Education: MDT clinic play an important role as a knowledge hub to patients and caregivers about the rare disease and its management. In general, there is a scarcity of information on rare diseases, including the natural history of the disease. It is therefore vital that patients and carers are well informed. Evidence from other chronic diseases [13] suggests the patient education empower patients to self-manage and coordinate their care.
- Research: The MDT clinics offer the opportunity for patients to participate in research to better understand the natural history of the disease and ultimately develop new types of therapy.
- Ownership: patients felt they own their service and their voice is well heard in shaping their care pathway. They also felt they are empowered to make their own health choices.

### Care-givers

- Acquired sufficient knowledge and experience to manage a complex rare disease. When disease prevalence is less than 1:1,000,000 it is obvious that most specialist physicians may not have seen the condition when they encounter patient with ultra-rare disease and a single or two patients might be the only patient they will see in their entire carrier. It is therefore bound to be a knowledge gap and lack of experience in managing rare patient’s complex need and care-givers feel inadequate and ultimately impact on the quality of care they provide. A recent global study looking at Hepatitis C management identified poor patient care was strongly associated with lack of knowledge of treating Physician [14]
- Shared knowledge of experts by having several specialists under one roof. Specialists learned from each other and able to communicate effectively by having pre and post clinic MDT meetings (rather than email/letter or scheduled meetings).
- Patients attended all their clinic and test appointment. It is well recognised that patient non-attendance (DNAs) in chronic disease have an enormous impact on the healthcare system in terms of cost and waiting time, significantly adding to delays along the patient pathway. Our patients 100 % clinic attendance allow us for efficient running of clinics, increase productivity, reduce costs and improve care [15].
- Able to undertake research in the particular rare disease and improve not only the care of our own patient but the AS community at large [16–18]. Pooling all the UK and international patient allow us to acquire sufficient number of patient to undertake several large scale research initiatives in including European patient registry [19].

### Impact on patient life/society

The medical journey travelled by patients with a rare disease (and their families) from initial disease recognition or onset of symptoms to a final diagnosis and treatment may involve serial referrals to several specialists and a plethora of often invasive and repetitive tests. This odyssey can be long and not fully understood by medical professionals. This has serious consequences for the health of patients with delayed diagnosis and suboptimal therapy. The hidden cost of treating and caring for patient with rare disease is substantial. For example patients attending a dedicated MDT clinic made fewer visits compared to the standard care, reducing the cost related to their healthcare. In addition, AS has huge burden on family, friends, health and social service requiring disproportionately large resource from tax payer. On the other hand, if patients are treated optimally, he/she can therefore be a net contributor to the national economy rather than beneficiaries.

#### Limitations and further analysis

Several limitations require attention. First, the overall sample size is small. We reached out to all UK patients and were able to recruit a quarter of the UK cohort. Second, comparable data is scarce in rare and ultra-rare disease and unable to fully evaluate our MDT service. One would hope this is the first of many best practice sharing studies in rare and ultra-rare disease care. Third, although the large bulk of the costing is accurate (direct personnel costs) other costing (indirect costs/follow-up costs locally/consumables) is based on national tariffs and educated assumptions and may under-estimate cost. This, however, will be the same pre and post MDT costing and hence direct comparison can still be made. Fourth, the MDT clinic was designed as a clinical service rather than for research and we were unable to capture fully clinical outcome measures prior to MDT clinic (2006) and hence our outcome measure was based on patient reported outcome.

## Conclusion

Despite the limitations of the study we believe the model of care we have outlined in this article provides a best practice for ultra-rare disease care. It is patient led, individually tailored to a complex need and at a cost that is comparable to the standard care. In addition, the MDT clinics have served as a referral centre for international patients and a nucleus in driving large-scale research in AS. Finally, AS patients’ centred and personalised care can serve as a paradigm for other rare and ultra-rare diseases.
